# Regulating genetic discrimination in Chinese employment: insights from thalassemia-related cases

**DOI:** 10.1093/jlb/lsag019

**Published:** 2026-06-04

**Authors:** Lingqiao Song, Weitong Zhang, Yann Joly

**Affiliations:** Centre of Genomics and Policy, Victor Phillip Dahdaleh Institute of Genomic Medicine, McGill University, Montreal, Canada; College of Philosophy, Law & Political Science, Shanghai Normal University, 100 Guilin Road, Shanghai, China; Centre of Genomics and Policy, Victor Phillip Dahdaleh Institute of Genomic Medicine, McGill University, Montreal, Canada

**Keywords:** Chinese law, discrimination in workplace, genetic discrimination, genetic information, group harm, lost in power

## Abstract

Genetic discrimination (GD) involves an individual or a group being negatively treated, unfairly profiled, or harmed, relative to the rest of the population, because of genetic characteristics. Research has examined GD mainly in insurance and employment, with growing attention to immigration, finance, forensics, and education. China, as a major biotechnology actor, has expanded genetic testing and supported rapid growth in gene-sequencing enterprises. These developments have also heightened concerns about GD, particularly in employment contexts. Since 2009, five employment-related GD cases have been reported in China. Two proceeded through judicial processes, while three became discussed social incidents. These cases share several features: all involved individuals carrying thalassemia-related gene mutations; all occurred in southern China, where populations exhibit genetic adaptations to hot, humid climates; and none reached a satisfactory resolution. Notably, all incidents arose within public-sector or quasi-governmental institutions that operate as extensions of state governance.

China’s legal framework contains significant gaps in addressing employment-related GD, creating risks of systemic exclusion for individuals with certain genetic traits. Because these cases occurred in the public sector, such exclusion restricts political representation. We recommend reviewing sector-specific labor and administrative regulations, classifying genetic information as sensitive personal data, and strengthening mechanisms to ensure employment opportunities.

## I. INTRODUCTION

The completion of the first human genome sequence in the 21st century marked a major milestone in our understanding of the human genetic code.^1^ With the emergence of second-generation sequencing, high-throughput technologies, and the integration of generative artificial intelligence, genomic research has entered a phase of rapid expansion.^2^

As a leading actor in biotechnology, China has achieved significant milestones in the field of genetic research. With the launch of national precision medicine initiatives and the growing availability of direct-to-consumer (DTC) genetic tests, genetics has become increasingly integrated into mainstream healthcare field.^3^ Genetic testing has become widely accessible online, allowing consumers to obtain information about their genetic code. In China, DTC genetic tests can now be easily purchased online through platforms such as Taobao for 499 Yuan (approximately 70 US dollars) without a prescription.^4^ Reports indicate that the DTC market in China is experiencing steady growth, and that a vast majority of users are willing to participate in research activities conducted by service providers.^5^ The increased accessibility of genetic information has amplified the risk for individuals to be negatively treated, unfairly profiled, or harmed, relative to the rest of the population, on the basis of actual or presumed genetic characteristics—a phenomenon commonly referred to as genetic discrimination (GD).^6^

Early studies on GD were primarily concentrated in North America, beginning in the 1970s.^7^ More recently, following the rapid advancement of genetic technologies in East Asia, research on GD in Asian countries has expanded over the past decade. For example, Hannah Kim et al. recently provided a socio-ethical and legal review of GD across multiple Asian countries.^8^ While there are commonalities between Asian countries, GD in China also exhibits distinct characteristics. Unlike in many other countries, where GD in the insurance sector is a prominent concern, instances of discrimination within the Chinese insurance industry are rarely reported.^9^^,^^10^ Moreover, collaborations between insurance companies and genetic testing companies are not uncommon. These partnerships may involve jointly designing products or offering bundled services that use genetic information to predict pharmacogenomic outcomes and the efficacy of specific medications.^11^ For instance, BGI Genomics, in collaboration with Taiping Property Insurance Company, Qianhai Reinsurance, and Chenxi Health Company, launched a health insurance product called Yixiangbao. The product provides coverage for the recurrence of certain types of cancer for customers who undergo genetic testing to detect circulating tumor DNA after purchasing the insurance.^12^ In contrast, five cases of GD in employment have been reported in China since 2009, partly due to gaps in the existing legal framework. However, research focusing on GD in the employment sector in China remains limited. This paper thus focuses on the emerging issue of GD in employment and is structured as follows: the first section outlines five reported incidents of GD in employment; the second section summarizes the common features of these cases and highlights underlying systemic discrimination issues; the third section examines China’s current legal framework regarding GD in employment, identifies institutional gaps revealed by these cases, reviews regulatory approaches adopted in other countries; and the fourth section proposes potential normative solutions to address GD in employment in China.

## II. METHODOLOGY

This study consists of a targeted literature review and a social media review to identify documented incidents of GD in employment. First, two researchers conducted comprehensive searches on legal databases including China Judgments Online, China Court Network, and PKULAW using the following keywords: labor disputes (劳动纠纷), employment discrimination(就业歧视), GD (基因歧视). This initial search yielded a total of 136 cases. Disagreements were resolved by a third researcher.

After reviewing all cases, only those involving discrimination related specifically to genetic mutations and occurring within the employment context were included. Ultimately, two cases met the inclusion criteria (see Table 1).

**Table 1:** Case comparison of GD in employment

Category	Case 1 (Zhou, Xie, and Tang)	Case 2 (Wang)	Case 3 (Lang Hao)	Case 4 (Li Yu)	Case 5 (Lin Fang)
Year	2009	2024	2010	2023	2025
Province	Guangdong	Fujian	Guangdong	Fujian	Fujian
Mutation	Thalassemia carrier	Thalassemia carrier	Thalassemia carrier	Thalassemia carrier	Thalassemia carrier
Position	Civil servants	Public-school teacher	State-owned enterprise, Audit Supervisor	Public-school teacher	State-owned daycare teacher
Cause (Legal Basis Alleged)	Administrative litigation; equal-employment rights	Administrative litigation; equal-employment rights	N/A	N/A	N/A
Litigation Result	The court dismissed the appeal and upheld the original judgment.	The court dismissed the appeal and upheld the original judgment.	—	—	—
Reason for Losing (Court Finding)	The refusal decision made by the Human Resources and Social Security Bureau is procedurally legal. The review of the appropriateness and validity of the Recruitment Standard is beyond the scope of the court’s decision.	The refusal decision made by the Siming District Bureau of Education is procedurally legal. The local regulatory document cited by the Bureau does not contravene higher-level laws.	—	—	—
Impact	This was the first legal case of GD in China and resulted in the revision of Guangdong’s medical examination regulations. However, the reforms have had only limited practical effect.	The existing Chinese legislation and judicial mechanisms are insufficient to resolve GD in the employment sector.	More unreported GD cases in employment ranging from civil servants to state-owned enterprises.	Triggered public calls to revise the *Civil Servant Recruitment Medical Examination General Standards* (Trial).	The Fujian Education Bureau prepared a proposal on resolving the ‘thalassemia’ issue.

**Table 2:** China’s norms in addressing GD and its deficiencies

Legal/regulatory instrument	Regulatory authority	Relevant provision(s)	Limitations
Constitution of the People’s Republic of China of 1982 (Revised in 2018)	National People’s Congress	Article 42: Citizens of the People’s Republic of China have the right and the duty to work.	Constitutional provisions shall not serve as a direct legal basis unless a sector-specific law implements them; thus equal employment rights under the Constitution remain nominal rather than practically enforceable.
Civil Code of the People’s Republic of China of 2021	National People’s Congress	Article 109: The personal liberty and dignity of a natural person is protected by law. Article 991: The personality rights of persons of the civil law are protected by law and should not be infringed upon by any organization or individual. Article 1034: The privacy of a natural person is protected by law. Privacy refers to a natural person’s private life, private space, private activities, and private information that he or she does not wish others to know. For the purposes of this Part, personal information refers to various information, recorded electronically or by other means that can identify a specific natural person either alone or in combination with other information, or reflect the activities of a specific natural person, including the natural person’s name, date of birth, identification number, biometric information, address, telephone number, and so on.	These provisions concerning human dignity and general personality rights are broadly framed and abstract, lacking specific clauses that directly regulate employment-related GD. Furthermore, the Civil Code does not explicitly include genetic information as a type of sensitive personal information.
Personal Information Protection Law of the People’s Republic of China of 2021	National People’s Congress Standing Committee	Summarized from Article 28: processing of ‘sensitive personal information’ (which may include health, biometric data, etc.) requires higher safeguards; separate consent for sensitive personal information; other articles (eg, Article 55) require impact assessments.	The law does not specify genetic information as a special type of sensitive personal information.
Labor Law of the People’s Republic of China of 1994 (Revised in 2018)	National People’s Congress Standing Committee	Article 12: Prohibits discrimination in employment on the basis of ethnicity, race, gender, or religious belief.	No explicit prohibition of GD in employment.
Employment Promotion Law of the People’s Republic of China of 2015	National People’s Congress Standing Committee	Article 3: The laborers shall have the right to equal employment and to choose jobs on their own initiative in accordance with law. The laborers seeking employment shall not be subject to discrimination on the basis of ethnicity, race, gender, religious belief, etc. Article 62: The laborers may lodge a lawsuit in the people’s court against those who commit employment discrimination in violation of the provisions of this Law.	No explicit prohibition of GD in employment.
Regulation on the Management of Human Genetic Resources of 2019 (Revised in 2024)	State Council of the People’s Republic of China	Article 9, Paragraph 2: The collection, preservation, utilization and external provision of China’s human genetic resources shall respect the right of privacy of human genetic resource providers, obtain their prior consent, and protect their lawful rights and interests.	Primarily targets research institutions and enterprises handling human genetic resources; does not cover the employment context or use of genetic information in hiring decisions.
Measures for the Administration of Health Insurance of 2019	China Banking and Insurance Regulatory Commission	Article 17: Except for family heredity history, health insurers may not base differential pricing on other genetic information or gene test data of the insured.	Applies only to the health-insurance sector; does not address employment or recruitment discrimination based on genetic information.
Notice on Adjusting Examination Standards for Relevant Items in the Civil Servant Recruitment Medical Examination of 2025	The General Office of the Organization Department of the CPC Central Committee and the General Office of the National Health Commission	I. Individuals with thalassemia (thalassemia gene carriers, silent types, and mild types) whose hemoglobin level is above 90 g/L shall be deemed qualified.	This standard is only applicable to civil servants and remains specific to one mutation type (thalassemia), thus lacking broader regulatory frameworks to address GD in other fields.
Notice on Issuing the Implementation Rules for Physical Examinations of Publicly Recruited Personnel in Guangdong Provincial Public Institutions (Trial) of 2010	Organization Department of the CPC Guangdong Provincial Committee, Department of Finance of Guangdong Province, Department of Human Resources and Social Security of Guangdong Province, and Department of Health of Guangdong Province	Article 3, Individuals with thalassemia who are able to perform normal work may be deemed qualified.	Region-specific regulation with limited binding effect, confined to public-institution recruitment within Guangdong Province and specific to one mutation type (thalassemia).
The General Standards for the Physical Examination of Public Institution Recruitment in Guangxi Zhuang Autonomous Region (Trial) of 2024	Department of Human Resources and Social Security of Guangxi Zhuang Autonomous Region and Health Commission of Guangxi Zhuang Autonomous Region	Article 3, Individuals with thalassemia (including gene carriers, silent types, and mild types) with hemoglobin levels above 90 g/L, and not affecting normal work, may be deemed qualified; if the state or relevant industry has other requirements or regulations, those shall apply.	Region-specific regulations with limited binding effect, confined to public-institution recruitment within the Guangxi Region and specific to one mutation type (thalassemia).

To supplement the judicial data, we conducted an additional search of Chinese social media platforms and online news sources, including Baidu, Sina, WeChat News, and Tencent, using the same keywords. This search produced 158 online articles. We included only GD incidents occurring in the employment context that were reported by authoritative media sources and provided specific information about the victims and sufficient supporting documentation. News reports and documents that did not describe specific cases, or that only discussed the fear of GD, general trends, or potential risks of GD, were excluded. Three additional cases met the inclusion criteria and were included in the present study (see Table 1).

## III. RESULTS AND DISCUSSION

### I‌II.A. GD in Employment from 2009 to 2025

All five cases are related to a specific genetic mutation of thalassemia. To contextualize the cases analyzed in this study, it is essential to first outline the genetic and diagnostic profile of thalassemia. Thalassemia is an autosomal recessive blood disorder characterized by reduced or absent synthesis of hemoglobin chains and is primarily categorized as α-thalassemia and β-thalassemia. The carriers discussed in this study are heterozygous individuals who possess a single pathogenic allele. These individuals are typically asymptomatic or present with only mild microcytic anemia and generally maintain normal health and working capacity.^13^

Despite the absence of severe clinical symptoms, carrier status can often be detected through routine blood tests, which often reveal reduced mean corpuscular volume and mean corpuscular hemoglobin. Such phenotypic indicators make carriers identifiable in routine screening contexts. Severe clinical manifestations occur primarily in individuals who inherit two pathogenic alleles.

Thalassemia carriers are particularly prevalent in southern China, a distribution historically associated with selective pressure from malaria. In some high-prevalence regions such as Guangdong and Guangxi, carrier rates have been estimated at approximately 9 per cent–11 per cent or higher.^14^ While definitive diagnosis requires molecular genetic testing, carrier status is frequently identified during routine pre-employment physical examinations based on hematological indices.^15^

The 2009 ‘first case of genetic discrimination’ involving thalassemia carriers in Foshan, Guangdong, marked the beginning of the judicialization of employment-related GD in China. Zhou, Xie, and Tang all performed well in both the written and interview stages of their civil service examination.^16^^,^^17^ According to the *Civil Servant Recruitment Medical Examination General Standards* (Trial) (hereafter referred to as ‘*General Standards*’), individuals with blood disorders were ineligible for employment. As carriers of the thalassemia gene, the three candidates were deemed medically unfit and consequently were not hired.^18^ They filed a request for administrative reconsideration against the Foshan Municipal Human Resources and Social Security Bureau. Following the failure of the administrative review, they pursued administrative litigation. In their lawsuit, the plaintiffs argued that their carrier status did not constitute a ‘hematological disease’ as stipulated in the *General Standards.* Accordingly, they petitioned the court to order the Bureau to certify them as medically qualified and to resume the formal assessment and recruitment process. However, the litigation proceeded through both first and second-instance courts, ultimately resulting in a ruling against them.^19^ On September 5, 2010, the Foshan Intermediate People’s Court, in its final judgment, held that the physical examination procedures were lawful and that carrying the thalassemia gene fell within the category of hematological diseases as defined in the *General Standards*. The court dismissed the appeal and upheld the original judgment.^20^

During the trial, the court issued a judicial recommendation to the Human Resources and Social Security Bureau, requesting an investigation into the employment of asymptomatic thalassemia carriers. The case prompted Guangdong Province to revise its medical examination regulations for public institutions in 2010, explicitly stating that asymptomatic thalassemia carriers should be considered qualified for employment if their condition does not affect work performance. However, the plaintiffs’ lawyer stated that the scope of this policy reform remains limited, and similar incidents continue to be reported in other provinces.^21^ Fifteen years after the first case of GD, a similar incident was reported in Xiamen, a neighboring city of Guangzhou, where the initial case occurred. In August 2024, a candidate, Wang, who ranked first in both the written and interview stages of the Xiamen Siming District primary and secondary school teacher recruitment, was denied employment after a pre-employment medical examination revealed that he was a carrier of the thalassemia gene.^22^

Wang filed an administrative lawsuit on the grounds of ‘employment discrimination’, arguing that the Siming District Bureau of Education had failed to exercise reasonable diligence and violated higher-level laws, thereby infringing upon his rights to equal employment and labor.^23^ The case was heard in the first instance by the Jimei District People’s Court and in the second instance by the Xiamen Intermediate People’s Court.^24^ Both courts held that Article 3 of the *Medical Examination Standards for Teacher Qualification Applicants in Fujian Province (2018 Revision)* (hereafter referred to as ‘*Standards in Fujian*’), did not constitute employment discrimination, nor did it violate provisions on personality rights and equal employment under the *Civil Code*, the *Employment Promotion Law*, or the *Labor Law*.^25^ In Chinese administrative litigation, lower-level regulatory rules such as the *Standards in Fujian* are classified as regulatory documents. Under Paragraph 2 of Article 148 of the Interpretation of the *Administrative Procedure Law*, courts have the authority to review such regulatory documents and decline applying them if they are found to conflict with higher-level national laws. Wang challenged the legitimacy of the *Standards in Fujian* on this basis. However, the courts concluded that the *Standards in Fujian* did not contravene any higher-level statutes, and affirmed the legality of the regulatory document itself. The court therefore dismissed Wang’s claims.^26^

This case perpetuates the central controversy of the 2009 Foshan ‘thalassemia gene discrimination case’, whether civil service medical examination standards excessively restrict individuals who are gene carriers. The courts relied on the principles of ‘primacy of medical professional judgment’ and ‘legitimacy of administrative discretion’ as the main basis for their reasoning, thereby avoiding substantive review of the reasonableness of the examination standards.^27^

Beyond the two cases that have been formally filed to the court, many more carriers of the thalassemia gene continue to experience discrimination in employment, particularly in southern regions of China.^28^ We found that, from 2010 to 2025, three more cases have been reported in the news or on social media. (Shown in Table 1) In 2010, Lang Hao was refused a state-owned enterprise position due to genetic mutation of thalassemia.^29^ In 2023, Li Yu, a candidate in Xiamen, ranked 40^th^ overall for the position of elementary school Chinese teacher through the public recruitment written and interview examinations.^30^ In May, following a prenatal checkup that revealed information related to thalassemia, the Personnel Office of the Siming District Education Bureau required her to undergo additional genetic testing for the thalassemia gene. She was subsequently denied employment on the grounds of a ‘blood system disorder’ and deemed ‘unqualified’. Li Yu’s hemoglobin level during the prenatal examination was 130 g/L, within the normal reference range for adult women, and her carrier status would have been extremely difficult to detect without the genetic test results.^31^ This case also highlights a growing concern where employers obtain medical records through independent investigations or third-party reports and use them as a basis for dismissal of employment; such practice constitutes an infringement on the right to privacy and violates the *Personal Information Protection Law* (PIPL) regarding the processing of sensitive personal data. In 2025, another victim Lin Fang was terminated from her employment after her employer became aware that she carries Thalassemia gene.^32^

Many carriers who remain unreported may also experience discrimination like those of Li Yu and Lin Fang. Research indicates that this autosomal recessive genetic disorder is primarily regionally concentrated, with a high-prevalence belt spanning ten provinces, autonomous regions, and municipalities south of the Yangtze River: Guangdong, Guangxi, Hainan, Fujian, Yunnan, Guizhou, Sichuan, Hunan, Jiangxi, and Chongqing. To identify thalassemia gene carriers, some employers have unilaterally added genetic testing requirements to pre-employment medical examinations or rescinded employment offers upon learning of an applicant’s carrier status.^33^

### I‌II.B. GD in Employment: A Systemic Problem

Based on the two previously adjudicated legal cases and three widely reported social incidents, additional observations emerge within the Chinese context. First, the five cases exhibit pronounced regional and disease-specific characteristics. All occurred in southern China, where the climate is hotter and more humid than in other regions. Populations in this area exhibit a higher prevalence and combined carrier rate of α- and β-thalassemia gene mutations, which historically confer a selective advantage against environmental stressors such as heat and humidity. Individuals carrying these mutations typically display lower hemoglobin and oxygen levels, which may have conferred adaptive benefits in this environment.^34^

However, in modern society, with access to air conditioning and improved living conditions, this genetic advantage has receded and, in some circumstances, has become detrimental. Consequently, individuals carrying these genes now face increased social and health-related challenges, including stigmatization and discrimination.

Notably, all reported cases of employment-related GD in China have involved thalassemia, as it can be initially detected through routine blood tests and subsequently confirmed via genetic testing. This ‘high identifiability’ renders carriers particularly vulnerable to exclusion at employment entry points.^35^ In contrast, mutations such as BRCA1/2, associated with breast cancer or the CAG-repeat expansion in the *HTT* gene linked to Huntington’s disease are difficult to identify in carriers without genetic testing during the asymptomatic stage, making the risks of discrimination comparatively covert.^36^^,^^37^ In other words, thalassemia carriers, even without a genetic test, can be identified through simple blood test results and thus function as a ‘visible indicator’ for group-based GD due to their relative ease of detection. However, with the widespread clinical application of genetic testing, particularly the proliferation of DTC testing, it is foreseeable that future cases of employment-related GD could increasingly involve other genetic conditions.^38^

GD in employment can also give rise to intergenerational injustice. The heritability of genetic mutations extends the effects of discrimination across generations. Carriers not only face the deprivation of their own employment opportunities, but their children and grandchildren may also be subject to systemic exclusion due to carrying the same genotype.^39^ Indeed, β-thalassemia is an autosomal recessive genetic disorder, meaning that if both parents carry a mutated gene their child could be affected.^40^ When individuals pursue legal action despite limited prospects for success, their motivation often shifts from seeking individual redress to advocating for ‘intergenerational justice’. As Li Yu stated, ‘I am speaking not only for myself but also for my daughter’.^41^

The situation is further exacerbated by the fact that all five cases occurred in positions within the civil service (*Gōngwùyuán*)^42^ or quasi-civil service institutions (*Shìyè dānwèi*).^43^ These positions are characterized by ‘high social status, high salary, and high stability’, making them the preferred choice for university graduates. Public data indicate that approximately 60 per cent of undergraduate degree holders rank positions within the state system as their first choice.^44^ Intense competition has directly raised the screening thresholds for candidates, increasing the demand for quantifiable risk indicators, and employers could decide to include asymptomatic carrier status of genetic conditions, to their exclusion criterion.^45^

More critically, GD in employment often occurs in positions in the public sector. These positions are not ordinary jobs—they are associated with concrete public authority and play a central role in administering state functions in China.

When individuals who are carriers of certain genetic mutations are systematically excluded from these positions, they are effectively removed from key channels of public participation and decision-making. This creates a form of institutionalized exclusion, where an entire group is prevented from accessing stable, influential public-sector careers solely because of their genetic status.

In other words, individuals carrying certain identifiable mutations may gradually lose representation and influence in China’s public sphere.^46^ With the adoption of the *Notice on Adjusting Examination Standards for Relevant Items in the Civil Servant Recruitment Medical Examination of 2025* (hereafter mention as the *Notice of 2025*), thalassemia may no longer constitute a basis for discrimination; however, numerous other genetic diseases remain unaddressed.

Such exclusion not only diminishes their voice within authoritative institutions but also exacerbates their already disadvantaged social position. Ultimately, the loss of representation and power may further entrench structural inequalities for this population.

Furthermore, although these five cases occurred in the field of employment, the interaction between eugenic practices and implicit selection in marriage and family planning exposes individuals to reproductive discrimination, thereby perpetuating intersectional cycles of disadvantage. Reports indicate that premarital examinations in China have been associated with discriminatory practices against individuals with hereditary conditions.^47^ Although premarital medical examinations are not mandatory, Article 8 of the current *Maternal and Child Health Law,* a law whose past is intertwined with eugenics practice in China, requires that they include screening for serious genetic diseases, which increases the possibility for GD to occur in the context of marriages. Given the increasing accessibility of genetic tests in medical examination, GD in marriage could become a setting for societal exclusion of individuals with genetic mutations.^48^

### I‌II.C. China’s Legal Framework Applicable to GD (see Table 2)

As discussed above, both legal cases (2009 and 2024) ultimately resulted in rulings against the plaintiffs, with similar legal reasoning underpinning the decisions. Employment-related GD cases involving civil service recruitment typically enter the judicial review process through administrative litigation. In such cases, courts assess whether the specific administrative action complies with procedural requirements and has a lawful basis. That is, they evaluate the legality of the statutory provision(s) invoked and whether the public authority followed proper procedures, rather than examining the substantive legitimacy of the laws or regulations underlying the administrative action.^49^

In the 2024 case, the court held that the formulation process of the *Examination Standards for Teacher Qualification Applicants in Fujian Province (2018 Revision)* conformed to relevant laws and procedural requirements, and consequently dismissed claims seeking a substantive review of the reasonableness of the document.^50^ This case demonstrates that courts do not evaluate the reasonableness of requiring thalassemia gene testing itself; rather, their review is limited to the legality of the specific administrative action and its procedural compliance.^51^ In the 2009 case, the court determined that the non-hiring decisions made by the Foshan Municipal Human Resources and Social Security Bureau were lawful in both their legal basis and procedural execution, and therefore upheld the validity of the employment decisions.^52^

A review of the current legal framework for addressing GD reveals several flaws. The right to work, a fundamental constitutional right and obligation in China, is guaranteed under the *Constitution of the People’s Republic of China*, which provides for equal employment under Article 42.^53^ Yet, in the Chinese legal system, constitutional provisions cannot serve as a direct legal basis in judicial proceedings unless they are implemented through sector-specific laws. In other words, unless sectoral labor laws operationalize the constitutional provisions on equal employment, the constitutional guarantees remain largely nominal rather than practically enforceable.^54^

Within China’s labor law framework, discrimination on the basis of ethnicity, race, gender, or religious belief is explicitly prohibited, as reflected in the *Labor Law* (2018).^55^ However, there is no specific prohibition against discrimination based on genetic information. Moreover, while the *Measures for the Administration of Health Insurance*, include provisions aimed at preventing GD in private health insurance, comparable protections are absent in the *Labor Law.*^56^ While the *Employment Promotion Law* has curbed discriminatory practices to some extent, existing judicial precedents remain largely concentrated in areas such as gender and regional discrimination.^57^ Because genetic characteristics are not explicitly codified as a protected category within statutory provisions, courts frequently encounter significant challenges in determining the appropriate legal framework when assessing whether such cases constitute actionable employment discrimination.

Human genetic information is also regulated under the *Regulations on the Management of Human Genetic Resources* (2019). This administrative regulation primarily aims to prevent the misuse of China’s human genetic resources by foreign entities. Although Article 9 prohibits violations of personal privacy, it contains no provisions specifically addressing GD. As an administrative regulation governing academic institutions and research organizations using human genetic resources, its focus is not on preventing employment-related GD arising from the misuse of genetic information.^58^

In terms of personal information protection, GD may result from the misuse of genetic information following a privacy breach. Therefore, effective sector specific governance of genetic information, aimed at preventing discrimination, is a key approach to addressing this issue.^59^ China’s PIPL of 2021 provides general provisions regulating the use of personal information but does not include specific clauses concerning genetic information.^60^ Although the National Technical Standard GB/T 35273 classifies genetic information as sensitive data, thereby requiring compliance with regulations applicable to sensitive data, the PIPL does not explicitly define genetic information as sensitive.^61^ This lack of clarity in the PIPL leaves room for uncertainty regarding the protection of genetic information.

Recent provincial policies in Guangdong and Guangxi—regions with the highest incidence of thalassemia in China—initially introduced targeted provisions to mitigate this issue. Regional regulations issued in 2010 and 2024 explicitly recognized thalassemia carriers and individuals with mild or silent forms of the condition as eligible for employment in public institutions.^62^^,^^63^ Building upon these regional initiatives, a significant national-level milestone was reached in November 2025. The Organization Department of the Communist Party of China (CPC) Central Committee and the National Health Commission jointly issued the *Notice on Adjusting the Examination Standards for Relevant Items in the Civil Servant Recruitment Medical Examination*. The Notice explicitly provides that individuals with thalassemia—including gene carriers, silent types, and mild types—whose hemoglobin levels exceed 90 g/L shall be deemed medically qualified.^64^ This reform substantively rectifies the long-standing practice of conflating an asymptomatic carrier state with a pathological ‘blood system disease’ in public service recruitment.

However, despite these progressive developments, limitations remain. Although the 2025 Notice elevates protection to the national level, its jurisdictional scope is strictly limited to civil service recruitment, leaving the vast workforce in the private sector without explicit statutory protection. Furthermore, both the regional standards and the new national policy share a critical limitation: they are highly disease-specific, applying only to thalassemia mutations. As noted above, thalassemia is uniquely susceptible to detection because it can be identified through routine blood tests. With the decreasing cost and increasing accessibility of comprehensive genetic sequencing, individuals carrying other recessive pathogenic variants may increasingly become identifiable and exposed to similar risks of employment discrimination. Consequently, this patchwork regulatory approach, limited to particular diseases, regions, or occupational sectors, cannot substitute for comprehensive, cross-sectoral national legislation prohibiting GD.

### I‌II.D. A Practical and Feasible Way to Address GD: Legislative Amendments in Existing Laws

From a normative perspective, Joly et al. (2017) examined regulatory and policy frameworks addressing GD, identifying distinct approaches: human rights, ethical guidance, industry self-policy, sectoral legislation, moratorium, reliance on existing generic legal protection, and hybrid models including characteristics of more than one approach. These categories were identified from international comparative legal review.^65^ For example, the European Union’s GDPR is classified as sector specific protection by including genetic information as sensitive data in data privacy law; it provides a higher degree of protection to this information.^66^ In the USA, GD is addressed through another sector specific approach by the Genetic Information Nondiscrimination Act (GINA), enacted in 2008. It covers health insurance under Title II and employment under Title III.^67^ This law provides baseline protection in employment, but it does not extend to employers with less than 15 employees nor to life insurance, education, or immigration. GINA was implemented by the Equal Employment Opportunity Commission which publishes useful report documenting the process on a yearly basis.^68^^,^^69^

These normative approaches provide guidance for possible solutions to GD in China. First, a legislative review of existing sectoral laws and regulations represents a feasible strategy. The incorporation of specific provisions to prevent GD in the *Labor Law*, particularly in Article 12, would serve as a fundamental mechanism to ensure the constitutional right to equal employment. Additionally, Article 3 of the *Employment Promotion Law* should explicitly include provisions preventing discrimination based on genetic information.^70^^,^^71^

From a personal information protection perspective, genetic information should be explicitly classified as sensitive personal data under Article 28 of the *PIPL.* This would clarify that the provisions on sensitive personal data under the PIPL also apply to genetic information. Such measures would provide greater protection to genetic information. The existing mechanisms for sensitive information—including separate informed consent and Personal Information Impact Assessments—should also be applied to prevent the unlawful misuse of genetic information from the outset.^72^ Unlike the USA, where the Equal Employment Opportunity Commission (EEOC) oversees GD in employment, China currently lacks an effective enforcement mechanism to ensure equal employment. The establishment of a similar organization as the EEOC could help ensure the implementation of the *Labor Law* and the *Employment Promotion Law* in China. However, given that many reported cases of GD in China arise within the public sector, the effectiveness of an EEOC-like model would depend on whether the regulatory body possesses sufficient authority and institutional independence to investigate public employers.

## IV. CONCLUSION

The cases of GD in employment discussed here are unlikely to remain isolated incidents. Rather, due to the increasing accessibility of genetic testing and the involvement of public institutions and government-affiliated entities responsible for exercising state authority, these cases reflect a broader systemic issue. GD in the employment sector has the potential to perpetuate intergenerational injustice. Such systemic exclusion represents the most severe consequence of GD, not merely the infringement of an individual’s rights, but the sustained, legally unprotected disadvantage imposed across multiple generations under the current legal framework. The absence of explicit legal protection exposes vulnerable populations to more systematic and intergenerational discrimination.

Normative approaches to GD in employment adopted in other countries, provide valuable guidance for China. Legislative review of sectoral laws and regulations is necessary to ensure equal employment regardless of one’s genetic predispositions. Genetic information should be explicitly recognized as a special category of personal information. Furthermore, the establishment of a similar body as EEOC could help ensure the enforcement of labor protections against discrimination in China. More than 17 years after the first GD case, it is imperative to take action to prevent the ‘gene lottery’ from determining employment outcomes, thereby protecting both individuals’ livelihoods and their voice in society.

## Footnotes

1 National Human Genome Research Institute (NHGRI), *Human Genome Project Fact Sheet*, https://www.genome.gov/about-genomics/educational-resources/fact-sheets/human-genome-project (accessed Oct. 27, 2025).

2 Heena Satam et al., *Next-Generation Sequencing Technology: Current Trends and Advancements*, 12 Biology 997 (2023).

3 Lei Cai et al., *The Forty Years of Medical Genetics in China,* 45 J. Genet. Genomics 569–582 (2018).

4 Chen Zhu. *Demand for Direct-to-Consumer Genetic Testing Services in China and Its Implications for Precision Public Health - China, 2021*, 4 China CDC Weekly 715–719 (2022).

5 K. Kang et al., *Direct-to-Consumer Genetic Testing in China and Its Role in GWAS Discovery and Replication*, Quant. Biol. (2020).

6 B. Kaiser et al., *A Proposal for an Inclusive Working Definition of Genetic Discrimination to Promote a More Coherent Debate*, 56 Nat. Genet. 1339–1345 (2024).

7 I. M. Rutkow & J. M. Lipton, *Some negative Aspects of State Health Departments’ Policies Related to Screening for Sickle Cell Anemia*, 64 Am. J. Public health 217–221 (1974).

8 H. Kim et al., *Genetic Discrimination: Introducing the Asian Perspective to the Debate*, 6 NPJ Genom. Med. 54 (2021).

9 Runguo Wu et al., *The Effects of Private Health Insurance on Universal Health Coverage Objectives in China: A Systematic Literature Review*, 17 Int. J. Environ. Res. Public Health 2049 (2020).

10 Kim et al., *supra* note 8.

11 Maxwell Brodie & Yann Joly, *DTC Pharmacogenetic Testing and Health Insurance: Good for Health, Good for Business?*, SSRN (Apr. 2, 2024).

12 BGI Group, *A New ‘Gene + Insurance’ Model! BGI Jointly Launches Medical Insurance for Monitoring the Recurrence of Specific Cancers(基因 + 保险新模式!华大基因联合发布特定癌症复发监测医疗险)*, https://www.bgi.com/news/2022111301 (accessed Mar. 5, 2026).

13 A. Kattamis et al., *Thalassemia*, 399 Lancet 2310–2324 (2022).

14 Aiqi Cai et al., *Prevalence, Mutation Distribution, and Economic Burden of Thalassemia in China: A Systematic Review and Regional Analysis*, 83 Arch. Public  Health 92 (2025).

15 Anam Khan & Aseeb U. Rehman, Laboratory Evaluation of Beta Thalassemia (2023), in StatPearls (StatPearls Publishing, 2026), https://www.ncbi.nlm.nih.gov/books/NBK585044/ (accessed Mar. 24, 2026).

16 Quanlin Qiu, *Thalassemia Gene Carriers Argue against Discrimination*, https://www.chinadaily.com.cn/china/2010-08/12/content_11141879.htm (accessed Oct. 26, 2025).

17 Yiming Liu et al., *Candidate with Anemia Gene Rejected from Job Application, Sues Labor Bureau but Loses Final Appeal (考生携贫血基因被拒录后状告人社局终审败诉)*, https://www.chinacourt.cn/article/detail/2010/09/id/426086.shtml (accessed Oct. 26, 2025).

18 Quanlin Qiu, *Carriers of Anemia Gene Are Denied Civil Servant Jobs*, https://www.chinadaily.com.cn/china/2009-09/18/content_8707123.htm?utm (accessed Oct. 26, 2025).

19 Liu et al., *supra* note 17.

20
*Id.*

21
*Id.*

22 Ruixiang Liu, *No Impact on Daily Life, Yet an Impediment to Public Sector Entry: the Institutional Barriers for Thalassemia Carriers in Civil Service Recruitment (不影响生活却‘影响’考编，‘地中海贫血’基因携带者的进编之路)*, https://www.jinantimes.com.cn/news-7-4960748.html (accessed Nov. 15, 2025).

23
*Id.*

24 Ruixiang Liu, *This Year of Thalassemia Gene Carriers Fighting Back Against Genetic Discrimination (‘地贫’基因携带者反‘基因歧视’的这一年)*, https://www.jinantimes.com.cn/news-7-5012349.html (accessed Nov. 15, 2025).

25
*Id.*

26
*Id.*

27 Liu, *supra* note 22.

28 Kim et al., *supra* note 8.

29 Guangzhou Daily, *White-Collar Worker Denied Employment Due to Thalassemia Gene: Genetic Discrimination Spreading in Workplaces (白领因携带地贫基因求职遭拒 基因歧视蔓延职场)*, https://www.qingdaonews.com/content/2010-07/30/content_8442805.htm (accessed Oct. 27, 2025).

30 Liu, *supra* note 22.

31
*Id.*

32 Rui Ke, *People who are carriers of the thalassemia gene should not face discrimination in employment (‘地贫’基因携带者，就业不该受歧视)*, THE BEIJING NEWS, (accessed Sept. 25, 2025) at A02.

33 Guangzhou Daily, *supra* note 29.

34 D. Chen et al., *Molecular Epidemiology and Hematological Thresholds for αβ-Thalassemia: A Retrospective Cohort Study in the Huizhou Region of Southern China*, 30 Hematol. 1–11 (2025).

35 Khan & Rehman, *supra* note 15.

36 Sharlene Hesse-Biber et al., *Intersectionality, BRCA Genetic Testing, and Intrafamilial Communication of Risk: A Qualitative Study*, 16 Cancers 1766 (2024).

37 M. Pengo & F. Squitieri, *Beyond CAG Repeats: The Multifaceted Role of Genetics in Huntington Disease*, 15 Genes 807 (2024).

38 Michelle Fernandes Martins et al., *Direct-to-Consumer Genetic Testing: An Updated Systematic Review of Healthcare Professionals’ Knowledge and Views, and Ethical and Legal Concerns*, 30 EUR. J. Hum. Genet. 1331–1343 (2022).

39 U.S. Department of Labor, *Genetic Information and the Workplace*, https://web.archive.org/web/20070506224349/http://www.dol.gov/oasam/programs/history/herman/reports/genetics.htm (accessed Oct. 27, 2025).

40 Arielle L. Langer, *Beta-Thalassemia*, www.ncbi.nlm.nih.gov/books/NBK1426/ (accessed Dec. 17, 2025).

41 Liu, *supra* note 22.

42
*Gōngwùyuán* (公务员) refers to a civil servant or public sector employee in China, typically someone employed by government agencies at the national, provincial, or local level. Civil servants are usually subject to specific recruitment, examination, and employment regulations under Chinese law.

43
*Shìyè dānwèi* (事业单位) refers to a public institution or quasi-governmental organization in China that provides social services, education, healthcare, research, or cultural functions. These entities are state-funded or state-affiliated but are distinct from civil service (*gōngwùyuán*) positions and often operate under different employment and administrative regulations.

44 Hongbin Li et al., *Job Preferences and Outcomes for China’s College Graduates*, 258 The  China  Quarterly 529–547 (2024).

45 Zhong Wang et al., *Attitude Disparity and Worrying Scenarios in Genetic Discrimination—Based on Questionnaires from China*, 11 Healthcare 188 (2023).

46 Kim et al., *supra* note 8.

47 Margaret Sleeboom-Faulkner, *Eugenic Birth and Fetal Education: The Friction between Lineage Enhancement And Premarital Testing Among Rural Households In Mainland China*, 64 The  China  Journal 121–141 (2010).

48 Y. Joly et al., *Comparative Approaches to Genetic Discrimination: Chasing Shadows?*, 33 Trends  Genet. 299–302 (2017).

49 Shiling Xiao, *State-Centric Proportionality Analysis in Chinese Administrative Litigation*, 21 Intl. J. Const. Law 461–487 (2023).

50 Liu, *supra* note 22.

51
*Id.*

52 Liu et al., *supra* note 17.

53 The State Council, *Constitution of the People’s Republic of China*, https://english.www.gov.cn/archive/lawsregulations/201911/20/content_WS5ed8856ec6d0b3f0e9499913.html (accessed Nov. 6, 2025).

54 Jun Yu et al., *How do Chinese judges invoke the constitution? Analysis based on 1907 decisions*, 7 Intl. J. Leg. Discourse 281–321 (2022).

55 National People’s Congress Standing Committee, *Labor Law of the People’s Republic of China*, http://www.npc.gov.cn/zgrdw/englishnpc/Law/2007-12/12/content_1383754.htm (accessed Nov. 17, 2025).

56 China Banking and Insurance Regulatory Commission, *Measures for the Administration of Health Insurance (健康保险管理办法)*, https://www.gov.cn/zhengce/zhengceku/2019-12/04/content_5458542.htm (accessed Nov. 6, 2025).

57 The Supreme People’s Court, *Guiding Case No. 185: Yan Jialin v. Zhejiang Sheraton Resort Co., Ltd, Dispute over the Right to Equal Employment(指导案例185号:闫佳琳诉浙江喜来登度假村有限公司平等就业权纠纷案)*, https://www.court.gov.cn/shenpan/xiangqing/364691.html (accessed Mar. 5, 2026).

58 Lingqiao Song et al., *Statistic Tells: The Regulatory Pendulum of Permit Trajectories in China’s Genetic Governance (2021–2024)*, 16 Front. Genet. 1611003 (2025).

59 Carolyn Riley Chapman et al., *Genetic Discrimination: Emerging Ethical Challenges in the Context of Advancing Technology*, 7 J Law  Biosci. lsz016 (2020).

60 The National People’s Congress of the People’s Republic of China, *Personal Information Protection Law of the People’s Republic of China*, http://en.npc.gov.cn.cdurl.cn/2021-12/29/c_694559.htm (accessed Oct. 28, 2025).

61 State Administration for Market Regulation; Standardization Administration of PRC, *GB/T 35273–2020 PDF English*, https://www.chinesestandard.net/PDF.aspx/GBT35273-2020 (accessed Oct. 28, 2025).

62 Department of Human Resources and Social Security of Guangdong Province, *Notice on Issuing the Implementation Rules for Physical Examinations of Publicly Recruited Personnel in Guangdong Provincial Public Institutions (Trial)(关于印发《广东省事业单位公开招聘人员体检实施细则(试行)》的通知)*, https://hrss.gd.gov.cn/zwgk/xxgkml/bmwj/gfxwj/content/post_3911066.html (accessed Mar. 7, 2026).

63 Department of Human Resources and Social Security of Guangxi Zhuang Autonomous Region, *The General Standards for the Physical Examination of Public Institution Recruitment in Guangxi Zhuang Autonomous Region (Trail)(广西壮族自治区事业单位公开招聘人员体检通用标准(试行))*, http://rst.gxzf.gov.cn/zwgk/xxgkzcfg/xxgkgfxwj/t18380985.shtml (accessed Mar. 7, 2026).

64 Communist Party Members’ Website, *Notice of the Organization Department of the CPC Central Committee and the National Health Commission on Adjusting the Examination Standards for Relevant Items in the Civil Servant Recruitment Medical Examination(中共中央组织部办公厅 国家卫生健康委办公厅关于调整公务员录用体检有关项目检查标准的通知)*, https://www.12371.cn/2025/11/14/ARTI1763103456803256.shtml (accessed March 7, 2026).

65 Joly et al., *supra* note 48.

66 Intersoft Consulting, *Art. 9 GDPR – Processing of Special Categories of Personal Data*, https://gdpr-info.eu/art-9-gdpr/ (accessed Oct. 27, 2025)

67 National Human Genome Research Institute, *Genetic Discrimination*, https://www.genome.gov/about-genomics/policy-issues/Genetic-Discrimination?utm_source (accessed Oct. 28, 2025).

68 Mark A. Rothstein, *GINA, the ADA, and Genetic Discrimination in Employment*, 36 J Law  Med. Ethics 837–840 (2008).

69 U.S. Equal Employment Opportunity Commission, *Genetic Information Discrimination*, https://www.eeoc.gov/genetic-information-discrimination (accessed Oct. 28, 2025).

70 National People’s Congress Standing Committee, *supra* note 55.

71 National People’s Congress Standing Committee, *Employment Promotion Law of the People’s Republic of China*, https://www.cecc.gov/resources/legal-provisions/employment-promotion-law-of-the-peoples-republic-of-china (accessed Nov. 17, 2025).

72 Rogier Creemers et al., *Translation: Personal Information Protection Law of the People’s Republic of China – Effective Nov. 1, 2021*, https://digichina.stanford.edu/work/translation-personal-information-protection-law-of-the-peoples-republic-of-china-effective-nov-1-2021/ (accessed Nov. 6, 2025).

